# Do intentionality constraints shape the relationship between motor variability and performance?

**DOI:** 10.1371/journal.pone.0214237

**Published:** 2019-04-17

**Authors:** Tomás Urbán, Carla Caballero, David Barbado, Francisco J. Moreno

**Affiliations:** Department of Sport Sciences, Miguel Hernández University, Elche, Spain; Washington University in Saint Louis School of Medicine, UNITED STATES

## Abstract

The aim of this experiment was to assess if the previously supported relationship between the structure of motor variability and performance changes when the task or organismic constraints encourage individuals to adjust their movement to achieve a goal. Forty-two healthy volunteers (aged 26.05 ± 5.02 years) performed three sets of cyclic pointing movements, 600 cycles each. Every set was performed under different conditions: 1) without a target; 2) with a target; 3) with a target and a financial reward. The amount of performance variability was analysed using the standard deviation of the medial-lateral (ML) and anterior-posterior (AP) axes and the bivariate variable error. The structure of the variability was assessed by Detrended Fluctuation Analysis (DFA) of the following time series: the coordinate values of the endpoint in ML, AP axes and resultant distance (RD), the hand orientation and the movement time. The performance of the task constrained with a target, or a target and reward, required higher implication to adjust an individual’s movements to achieve the task goal, showing a decrease in dispersions and lower autocorrelation. Under the condition without a target, variability dispersion was positively related to autocorrelation of the movement values from ML axis and RD time series, and negatively related to the values from the hand orientation time series. There was a loss of the relationship between variability structure and performance when the task was constrained by the target and the reward. That could indicate different strategies of the participants to achieve the objective. Considering the results and previous studies, the relationship between variability structure and performance could depend on task constraints such as feedback, difficulty or the skill level of participants and it is mediated by individual constraints such as implication or intentionality.

## Introduction

In cyclic movements, even if the subject tries to exhibit consistency, every movement is different from the previous one. In other words, each movement is unique and unrepeatable. These movement variations, as a prominent feature of motor control, have been a rising topic of interest over recent decades. The conventional assumption that movement variability is a product of unstructured noise, superimposed on a deterministic signal, has been questioned by recent studies, which suggests that movement variability is structured and reveals specific details of the system dynamics [1–4]. Motor variations enable continuous exploration of the possible motor states and neuronal configurations that can lead to the desired state by trial-and-error learning, playing an important role in motor learning and the ability to adapt [5–8]. Thus, movement variability helps to exploit the degrees of freedom of the biological system [9–11], providing the ability to adapt to the constraints (organismic, task and environmental) that shape the individual’s behavior [12].

Several studies have focused on the relationship between the structure of motor variability and motor performance [13–17] or learning processes [5]. These works have found that high degrees of irregularity and/or low values of autocorrelation in movement variability are related to the capability of the motor behaviour to find the best movement solution, causing better performance and higher ability to adapt. Nevertheless, this relationship remains somewhat unclear. Some studies have shown data that do not support the aforementioned relationship, reporting greater degrees of irregularity in movement fluctuations associated with worse task performance [18–20]. In order to address this controversy, Newell and Vaillancourt [21] suggested that the relationship between variability structure and motor performance depends on the nature of both the intrinsic dynamics of the system and the task contraints [20,22].

Some studies have found that emergent movement coordination patterns are shaped by specific task and organismic constraints [17,18,22–25]. Under specific constraints, participants search for functional information from the environment in order to regulate performance behaviours and find the best solution to achive the task goal, increasing or reducing the number of adjustments they have to perform. For example, Kuznetsov and Wallot [17] investigated the effect of feedback on the structure of variability in a continous tapping task and found that an increased amount of feedback leads to a decrease in long-range autocorrelations of the time interval estimations, as well to a decrease in the amount of local fluctuations in the motor performance. Individual’s actions are directly related to the perceived environment and the role of perception and action is to re-organise intentional behaviours during interactions with key environmental and task constraints [26]. For instance, feedback availability is a physical constraint that could increase participant intentionality in achieving the task goal due to the reinforment provided by sucessful trials. In this sense, some authors have related feedback constraints and the intention of the performer to deal with those constraints with the changes on the structure of variability in time estimation [27], tapping [28] and walking tasks [26,29]. Dingwell and Cusumano [30], reported that the output variables that were not tightly regulated exhibited statistical persistence, but increased central control over these variables resulted in uncorrelated or anti-persistent time series. Likens et al. [31] postulated that the structure of variability is related to the enactment of control, reflected in long range autocorrelated variability, specifically in fractal scaling.

Chen et al. [27] found that participants in an entrainment task keeping the rhythm of a metronome displayed higher irregularity in performance and lower autocorrelated fluctuations when feedback was available. Similar findings were obtained by Washburn et al. [24], who analysed participants’ intentions and the effect of task constraints on the structure of variability during a rhythmic movement task in which participants were asked to adjust timing or movement amplitude. Their results indicated that in the absence of a reference stimulus or feedback, differences in intention did not affect the variability structure. Nevertheless, when the stimulus was present, the variability of the movement displayed more random fluctuations and this behaviour was more prominent in the corresponding task dimension (timing or amplitude movement) the participant intended to control. Intention interacted with available task constraints to produce even greater random variations when the task dimension constrained by the stimulus was also the dimension the participant intended to control. These findings suggest that any intentional constraint is dependent on the concurrent existence of external constraints [24].

Previous studies have supported that there is an influence of task and organismic constraints on the relationship between the structure of variability and motor performance. However, direction or intensity of the influence, or interactions between constraints have not been well established. In a previous work carried out by Caballero et al. [25], it was found that the relationship between variability structure and performance in balance tasks appears with different prevalence depending on constraints like the difficulty of the task or whether participants received feedback of their performance in real time. When participants had to perform a day-life activity (e.g. standing still on a stable support) and biofeedback was not provided, a relationship between scaling behaviour and balance performance was not found. In that experiment, it was observed that participants displayed a behaviour similar to a fixed-point attractor when biofeedback was available, characterized by stationary fluctuations around a point in space state [32]. Nevertheless, participants explored the oscillatory COP dynamics [21] without biofeedback in the least challenging conditions. In this sense, when the task is not constrained by an explicit target, they could feel free to explore diverse options to accomplish the task, exhibiting a more disperse and non-stationary variability, or they could choose to repeat and maintain a predetermined movement around an implicit criterion. Under this situation it is expected that the relation between variability structure and performance appears. On the other hand, when the objective of the movement is explicit and participants have the intention to adjust to that objective, differences between participants’ strategies are reduced and the relation between variability structure and performance diminishes or disappears. However, despite the intentional and cognitive activity undoubtedly involved in performing everyday actions [26], the role of intentionality or participant implication during the execution of the tasks is still unknown. Therefore, the main aim of this study was to identify to what extent task and organismic contraints can regulate individual motor variability. Moreover, we investigated if the fluctuations exhibited in the task can reveal the degree to which the participant is constrained and if intentions actually act like an organismic constraint.

To achieve these aims, we manipulated the absence/presence of a spatial target and the presence of a reward in a cyclic pointing task. We analysed two global dimensions of motor variability (its amount and its structure) in order to analyze emergent movement dynamics. According to the literature, we hypothesized that the presence of a target in a cyclic task [33–35] and participants’ commitment in hitting the centre of the target would produce a shift in performance fluctuations towards less autocorrelated variability [17]. The reduction in long-range autocorrelation of variability will be identified with a stationary distribution of the pointing-endpoint positions around the target (i.e. a fixed-point attractor) revealing that an external constraint will guide participants to increase their intention of adjusting their pointing actions to accomplish the task. The experiment also has the goal of adding to the literature by showing that the presence of a constraint will specifically affect the variability structure of the variables constrained. And finally, we hypothesized that the constrained behaviour of participants would lead to a loss of the relationship between variability structure and performance.

## Methods

### Participants

Forty-two healthy volunteers (10 females, 32 males; aged 26.05 ± 5.02 years; right-handed) took part in this study. Participants were informed about the procedure, the tasks to be performed and the aim of the study. All participants signed an informed consent in accordance with the ethical guidelines of the host institution. The experimental procedures used in this study were in accordance with the Declaration of Helsinki and were approved by the institutional review board (Office for Projects Evaluation, OEP) of the host institution. The individual in this manuscript has given written informed consent to publish these case details.

### Instrumentation or apparatus

The kinematics to determine the accuracy of the pointing movement performed by participants were recorded using a 3D position sensor (8" Stylus) of Polhemus Liberty system (Polhemus Vermont, USA). Movement data were collected with a frequency of 240 Hz. Frequency of the movement, set at 1 Hz, was facilitated by a computerized virtual metronome.

### Procedure and design

**Fig 1:**
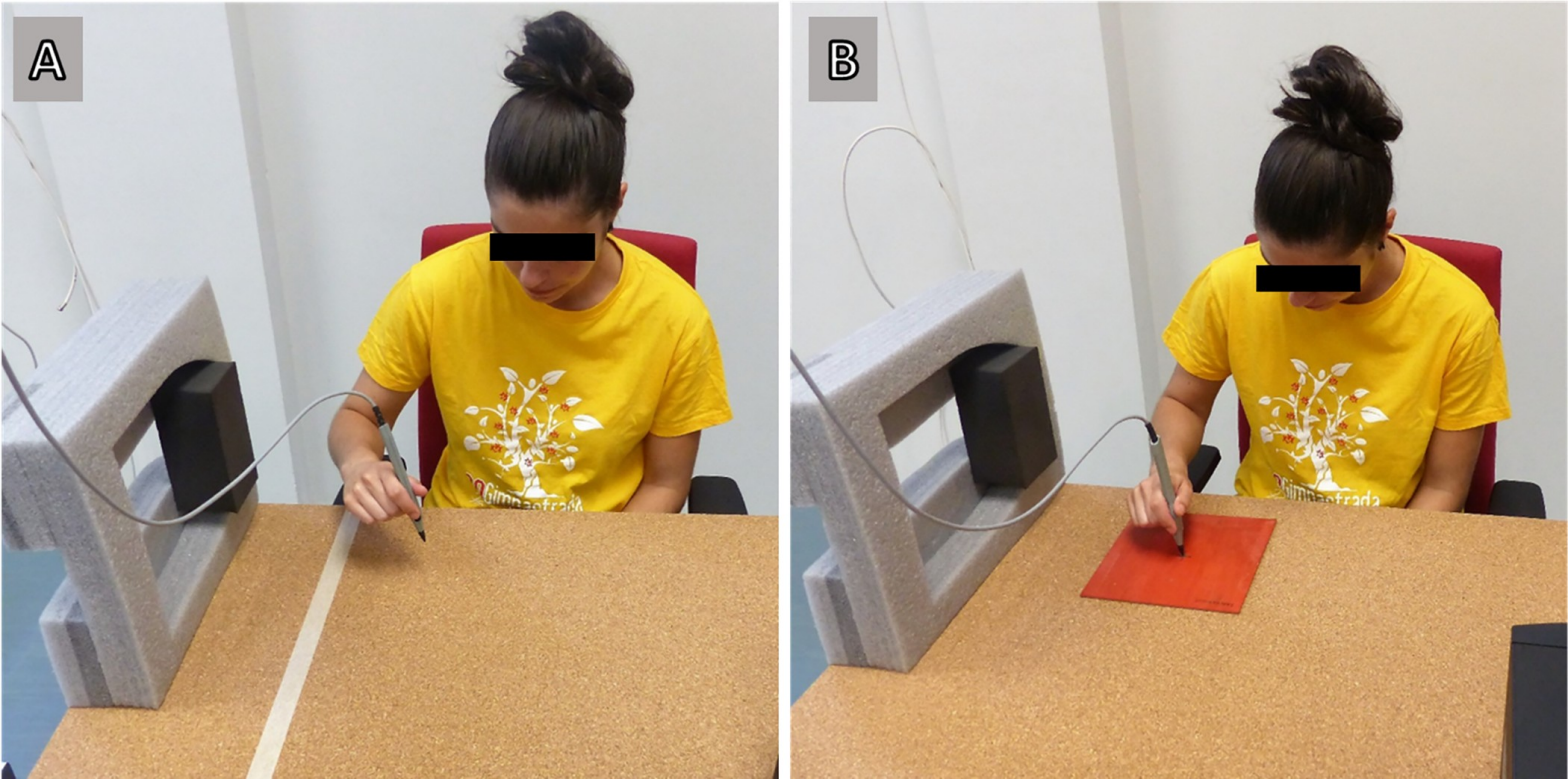
Experimental set up: A. Condition without a target, B. Condition with a target.

During the test, participants were comfortably seated in an adjustable ergonomic chair. They were located facing the table with the right shoulder aligned to the centre of the table, holding the forearm parallel to the floor without support. Throughout the task, participants maintained the arm next to the body with the elbow at 90 degrees (Fig 1).

The experimental undertaking performed was a pointing task in which participants were holding a stylus vertically with the tip resting on the surface. From this position, participants were told to lift the stylus and lead their hand toward a side to touch with the back of the hand one foamed vertical support located 20 cm to the right of the starting point. After touching on the vertical support, the participant was told to take the stylus back to the starting point to hit on the surface. The pointing movement was repeated cyclically until 600 cycles were performed. One cycle was considered from the starting point (when the tip of the stylus impacted on the surface) until the next one.

The frequency of the cycles was set up externally at 1Hz by a metronome.

Participants carried out three sets of pointing movements with 10 minutes of rest between sets. The task was performed under three different task constraints:

1) Condition without a target: participants started the task with the stylus tip on the table and were asked to point freely on the table with the only requirement of passing a line located 20 cm from the vertical support in order to perform a minimum displacement.

2) Condition with a target: participants aimed each movement at a target (displayed as a cross formed by two 2 cm long lines drawn on the surface). The point at which the lines crossed was considered as the centre of the target and it was located in front of the participant, 15 cm from the edge of the table and 20 cm from the vertical support.

3) Condition with a target and a reward: A monetary reward was offered to the participants to motivate them during the performance of the task [30–32]. Participants were instructed that they would be punished by decreasing the amount of the reward according to their accuracy. All participants knew the amount of money they could earn before starting the task, and they were informed that this amount would be reduced depending on the accuracy of their performance. The decrease in the amount of money was computed by a mathematical algorithm so that the participant never reached a value of zero during the trial.

In the first two conditions (with and without a target) participants did not receive any information about accuracy or movement time. However, in the third condition, participants received feedback about the amount of money they had remaining every minute. The order of the three conditions was counterbalanced between participants.

### Data analysis and reduction

Different data time series from the movement fluctuations during the pointing task were used to assess performance and movement variability. The coordinate values (in ML and AP axes) and hand orientation (computed by azimuth, elevation and roll of the stylus) at the endpoint position were collected for each cycle. The resultant distance time series to the midpoint (i.e. average value of each participant) (RD), combining AP and ML axes, and the movement time (MT) were also computed for each cycle.

### Measures of variability magnitude

In order to analyse the amount of performance variability, standard deviation of the medial-lateral (MLV) and anterior-posterior (APV) axes and the Bivariate Variable Error (BVE) of coordinate time series were computed. BVE was calculated as the average of vector distance magnitude (mm) of the endpoints position from the participant’s own mean endpoint position [36].

### Measures of variability structure

The coordinate values of the endpoint in ML and AP axis, RD, hand orientation (azimuth, elevation and roll) and MT of each cycle were measured. All data from the 600 repetitions were treated as time series and system dynamics were addressed through the variability structure analysis. Structure variability has been referred to as a functional feature, revealing movement dynamics and playing a fundamental role in coordination of perception and action [37,38].

Long-range dynamics of variability were analysed through autocorrelation of the time series, computed by Detrended Fluctuation Analysis (DFA) [39]. DFA analyses long-range correlations of signal using a parameter known as the scaling index, α [39]. This index identifies the extent to which proceeding data are dependent on previous outcomes, indicating the presence of long-term correlation within a time series [40]. These properties may show different values of α-DFA that indicate the trend of the time series. For instance, α-DFA > 0.5 implies persistence of the time series, i.e., the trajectory tends to continue in its current direction and values of α-DFA < 0.5 indicate anti-persistence, i.e., the trajectory tends to return from where it came [41]. In order to reduce error incurred by estimating α-DFA, the slope of α-DFA was obtained through a window size 4 ≤ *n* ≤ *N*/10 to maximize the long-range correlations [42].

### Statistical analysis

Shapiro-Wilk test was performed to evaluate the normality of variables. One-way repeated measures ANOVA was carried out to analyse the effects of the experimental conditions on the performance (amount of variability) and the structure of variability displayed by participants during the pointing task. Post-hoc pairwise comparisons between different conditions were conducted using a Bonferroni adjustment. The effect size was calculated based on the partial eta squared ($\eta_{p}^{2}$) to identify the proportion of the overall variance attributable to the factor. The values above 0.64 were considered strong effect, about 0.25 were considered moderate and ≤ 0.04 were considered small effect [43]. Finally, Pearson’s correlations coefficient was calculated to determine the relationship between variability dynamics (DFA) and performance (SD and BVE) during the execution of the different task constrains. The correlation levels were classified as excellent (0.90–1.00), high (0.75–0.89), moderate (0.74–0.50), slight, (0.25–0.49) and uncorrelated (< 0.25) [44]. Statistical tests were made using IBM SPSS Statistics 22.0.0.0. The alpha value of significance effect was established at 0.05.

## Results

The results regarding the endpoint dispersion obtained from participants in the task without a target were considered as a baseline to compare changes in the three task conditions. The one-way repeated measures ANOVA showed significant differences among the three experimental conditions (Table 1). Pairwise comparison between conditions revealed higher dispersion of endpoint position in the condition without a target while the lowest dispersions were found in the conditions with a target and a reward, showing a significant decrease of variability (MLV, *F*_1,41_ = 25.696, *p* < 0.001, $n_{p}^{2}$ = 0.385; APV, *F*_1,41_ = 15.957, *p* < 0.001, $n_{p}^{2}$ = 0.280; BVE, *F*_1,41_ = 30.554, *p* < 0.001, $n_{p}^{2}$ = 0.427).

**Table 1:** Average values of variability (mean ± SD.) in each pointing task condition and post-hoc pairwise comparisons among the three experimental conditions using Bonferroni adjustment.

Variability	Without target	With target	With target and reward	F	*p*	$\eta_{p}^{2}$
**MLV (cm)**	0.781 ± 0.715	0.153 ± 0.044 ^A^	0.117 ± 0.283 ^A^^B^	35.167	< 0.001	0.462
**APV (cm)**	0.627 ± 0.467	0.107 ± 0.026 ^A^	0.087 ± 0.023 ^A^^B^	54.503	< 0.001	0.571
**BVE (cm)**	0.853 ± 0.724	0.152 ± 0.042 ^A^	0.115 ± 0.025 ^A^^B^	42.597	< 0.001	0.510

^A^ Significant differences according to the condition without target.

^B^ Significant differences according to the condition with target.

MLV = Medial-lateral variability; APV = anterior-posterior variability; BVE = Bivariate Variable Error.

Long-range autocorrelation values of the time series of movement variability data were also analysed through repeated ANOVA measures, showing significant differences among conditions. Regarding the coordinate values in AP and ML axes and RD, post-hoc analysis displayed significant differences in long-range autocorrelation values between the condition without a target and both conditions with a target. In the condition with a target and a reward, a slight decrease of α-DFA values was found compared to the condition with a target, but the decrease was only significant in the AP axis (*F*_1.41_ = 8.952; *p* = 0.005; $n_{p}^{2}$ = 0.179). All values of α-DFA under the conditions without a target remained close to 1, while participants displayed α-DFA values close to 0.5 under the conditions with a target. In contrast, long-range autocorrelation values of hand orientation remained close to 1 in all conditions. Even so, post-hoc analysis also showed significant differences in long-range autocorrelation values between the condition without a target and both conditions with a target, although the trend was opposed (Table 2). The presence of a target increased α-DFA values compared with no target (Azimuth: *F*_1.41_ = 15.760; *p* < 0.001; $n_{p}^{2}$ = 0.278; Rotation: *F*_1.41_ = 4.413; *p* = 0.042; $n_{p}^{2}$ = 0.097) and they were even higher when the reward was added (Azimuth: *F*_1.41_ = 5.498; *p* = 0.024; $n_{p}^{2}$ = 0.118; Rotation: *F*_1.41_ = 8.943; *p* = 0.005; $n_{p}^{2}$ = 0.179).

**Table 2:** Average values (mean ± SD) in each pointing task condition of DFA of movement variability variables and post-hoc pairwise comparisons among the three different conditions using Bonferroni adjustment.

DFA of time series	Without target	With Target	With target and reward	F	*p*	$\eta_{p}^{2}$
**ML**	0.887 ± 0.234	0.475 ± 0.098 ^A^	0.447 ± 0.101 ^A^	112.599	< 0.001	0.733
**AP**	0.985 ± 0.232	0.571 ± 0.115 ^A^	0.509 ± 0.101 ^A^^B^	111.712	< 0.001	0.732
**RD**	0.957 ± 0.243	0.502 ± 0.108 ^A^	0.514 ± 0.081 ^A^	114.957	< 0.001	0.737
**Azimuth**	0.939 ± 0.155	1.06 ± 0.154 ^A^	1.13 ± 0.191 ^A^^B^	17.389	< 0.001	0.298
**Elevation**	1.03 ± 0.168	1.09 ± 0.188	1.16 ± 0.151 ^A^	5.912	0.004	0.126
**Rotation**	0.978 ± 0.197	1.06 ± 0.158	1.14 ± 0.188 ^A^^B^	11.394	0.002	0.217
**MT**	0.319 ± 0.254	0.271 ± 0.200	0.297 ± 0.242	0.743	0.479	0.018

^A^ Significant differences according to the condition without target.

^B^ Significant differences according to the condition with target.

ML = Mediolateral axis; AP = Anteroposterior axis; RD = Radial Error; MT = Movement Time.

On the other hand, MT showed no differences among any of the conditions performed with α-DFA values close to 0.3 in all conditions. The results of autocorrelation α-DFA values in every condition are shown in Table 2.

Under the condition without a target, variability dispersion from ML axis (MLV) was also positively related to long-range autocorrelation of movement variability values from ML axis and RD time series, and negatively related to the values from azimuth and roll orientations (Table 3). Variability dispersion from the AP axis (APV) was positively correlated with all long-range autocorrelated variables computed over AP, ML and RD time series. BVE was positively correlated with all long-range autocorrelated variables computed over AP, ML and RD time series but negatively correlated with the values from azimuth and roll orientations.

**Table 3:** Pearson product moment correlation coefficient calculated between error variability and movement variability, in each pointing task condition.

* *	* *	*MLV*	*APV*	*BVE*
***Without target***	***DFA_ML***	*0*.564**	*0*.502**	*0*.574**
	***DFA_AP***	*0*.311*	*0*.502**	*0*.389*
	***DFA_RD***	*0*.435**	*0*.580**	*0*.548**
	***DFA_Azim***	*-0*.436**	*-0*.*302*	*-0*.454*
	***DFA_Elev***	*-0*.*263*	*-0*.*048*	*-0*.*217*
	***DFA_Roll***	*-0*.400**	*0*.*000*	*-0*.306*
***With target***	***DFA_ML***	*-0*.*058*	*0*.*110*	*0*.*022*
	***DFA_AP***	*-0*.*016*	*-0*.*209*	*-0*.*133*
	***DFA_RD***	*-0*.*184*	*-0*.*074*	*-0*.*164*
	***DFA_Azim***	*-0*.*120*	*-0*.*017*	*-0*.*010*
	***DFA_Elev***	*0*.*205*	*0*.*120*	*0*.*099*
	***DFA_Roll***	*-0*.*202*	*0*.*075*	*0*.*022*
***With target and reward***	***DFA_ML***	*0*.*075*	*-0*.*102*	*-0*.*006*
	***DFA_AP***	*0*.*139*	*0*.*084*	*0*.*128*
	***DFA_RD***	*-0*.*207*	*-0*.*143*	*-0*.*215*
	***DFA_Azim***	*-0*.*232*	*-0*.*127*	*-0*.*292*
	***DFA_Elev***	*-0*.*017*	*-0*.*015*	*-0*.*053*
	***DFA_Roll***	*-0*.*064*	*-0*.*018*	*-0*.*111*

Regarding the conditions with a target and a target and reward, variability dispersion variables and long-range autocorrelation variables did not show any significant correlation. The results of the correlations for every condition are shown in Table 3.

## Discussion

Variability has been approached from different perspectives which have placed it at apparently opposite ends, from being considered as a limiting factor [45] to appearing as a necessary condition for the functionality of the system [2,12]. Traditionally, variability has been assessed as the amount of dispersion around a central criterion. Nevertheless, the use of nonlinear tools has boosted the study of control of human movement, allowing for the detection of changes of variability dynamics over time [3,46,47]. The relationship between variability and motor performance has generated numerous studies in recent years attempting to analyse this relationship in different tasks [13,16,17,48,49]. Nevertheless, the direction of this relationship currently seems to be unclear and it is still under discussion in many scientific forums. The main aim of this experiment was to assess if motor variability structure changes when tasks and organismic constraints encourage the individual to adjust his/her movement to achieve a goal during a cyclic pointing task. The performance of the pointing task without a target showed a higher amount of variability compared to the other two conditions and showed α-DFA values close to 1 for both coordinate and hand orientation data time series. The autocorrelation values shown in this situation could suggest a self-organized behaviour of an unconstrained system [50]. Previous researchers have proposed that α-DFA values close to 1 (frequently referred as pink noise or 1/f noise) is a general property of “interaction-dominant” systems [38,51]. That is, the activity of any unit of the system is functionally dependent on the activity of the other units. Van Orden et al. [23] proposed that the scaling of an observed behaviour reflects the degree to which the component processes have been constrained to operate as a single coordinated system. Thus, values of α-DFA close to 1 result when a balance of strong, rigid task constraints and more flexible, participant-enacted constraints exist to limit the degrees of freedom and sustain the task goal. During the first experimental situation, participants were not directed to point to any target any farther than to execute a minimum movement amplitude to normalize inter-participant data. This situation is compatible with a balance of voluntary and involuntary sources of motor control [23].

The presence of a spatial target caused a decrease in the amount of variability in the endpoint of the movements. This reduction in outcome variability reveals the effect of the target as a task constraint acting like an attractor of the movement. Participants exposed their intention to adjust their movement to a specific point in space in each cycle. This situation led to a modification of the variability structure of coordinate values time series, departing from α-DFA values close to 1 to less autocorrelated values close to 0.5. These results are similar to findings presented in previous studies in which participants received an external adjustment criterion, either temporal or spatial, and their movement showed a less correlated variability structure [16,17,27,28,52]. Following the previous rationale, the target in the aiming movement would act as a rigid constraint, shifting the movement outcome to continuous variations around the target and showing a less autocorrelated variability structure. Values of α-DFA around 0.5, related to white noise fluctuations, have been identified with the prevalence of rigid task constraints, causing the reduction of voluntary actions [23,24].

The application of rewards in addition of the presence of the target leads the participants to show an even lower amount of variability. The reduction in variability dispersion (decrease in RD from target condition to reward condition) correlated with the values of RD exhibited by participants in the target condition without reward (r = -0.832; p < 0.001). That is, participants with higher values of dispersion reduced it in a larger magnitude when the reward was introduced. This behaviour is compatible with an extra effort exhibited by participants in the intention to adjust their movement to the target when reward was added to the completion of the task. In this situation, for the coordinate values time signal, the autocorrelation of the variability showed even lower values, mainly in AP axis. The addition of a reward regarding the precision of the movements to the target, increased the power of the organismic constraint, that is, the participants’ implication in getting the target and, therefore, showing even less autocorrelated movement variations. The participants who exhibited higher values of autocorrelation in the situation with a target, farther from 0.5 α-DFA values, reduced these values to a greater extent when a reward was introduced (r = -0.682; p < 0.001). It would be reasonable to think that participants that exhibited higher autocorrelation values were less committed to the task or they were less constrained by the presence of the target. Supporting this assumption, we found a moderate correlation between α-DFA values in the situation with target and the reduction in RD from the situation with target to the situation with a target and a reward (r = -0.327; p < 0.05). Participants with higher α-DFA, farther from 0.5 values, showed not only a reduction in that value but also a greater reduction in fluctuations around the target when a reward was added. Therefore, the long-range autocorrelation of movement variability displayed by participants could reflect to what extent they were committed to adjusting their movement to an external reference, like the target. This is in accordance with Washburn et al. [24] results, which support the idea that participants’ intentions interacted with available task constraints (the target in this case) to produce an even greater shift toward random variations.

In this sense, correlational analysis showed a relationship between the structure of variability and performance when task was not constrained by the target, but this relationship disappeared when a target was added. Without a target, participants who displayed a lower amount of variability showed coordinate time series that were less autocorrelated. Some studies have indicated that lower autocorrelation values reflect a higher number of motion adjustments performed by participants [5,53,54]. In the situation without a target there was no target to adjust the tip of the stylus, but these results could reflect that some participants applied a coordination strategy based on maintain an “imaginary” target that served them as a reference for where to end their movements in every cycle. Those participants would display a lower amount of variability and more autocorrelated coordinate time series, indicating that they were performing a higher number of adjustments to touch the foam surface in the same place than those who did not use a personal reference.

These results support again the idea that the constraints have an effect on the variability structure of the task parameter constrained which the participant is trying to control, and this effect has an influence on the relationship between performance and variability dynamics. In fact, in the conditions with a target and with a reward, there was no relationship between performance and variability dynamics. Some studies have presented results along this line, finding a stronger correlation between performance and variability structure in unconstrained conditions [25,49]. It seems that the specific relationship shown between the dynamics of variability structure and performance depends on the task constraints [20–22,55]. The effect of different constraints modifies the relationship between the dynamics of variability and performance that exists when an unconstrained system shows a self-organized behaviour. Depending on what constraint is manipulated the modification of the relationship will be different.

The presence of the target and the application of a reward did not have the same effect over the hand orientation time series. The α-DFA values of the hand orientation remained close to 1 despite the target presence. Furthermore, α-DFA values in this situation (i.e. target and reward) were slightly but significantly higher than α-DFA values in the situation without the target. Those α-DFA values were subtlety higher than 1 indicating that hand position displayed a nonstationary behaviour with anti-persistent increments [56]. These findings support the idea that the effect of the task constraints is dependent on the task dimension the participant is trying to control. Washbourn et al. [24] explained that when constraints are present for a given task dimension, there is a shift in α-DFA values from the region associated with pink noise (α-DFA = 1) toward that related to white noise (α-DFA = 0.5) for that task dimension. In this study, the target caused a greater control over the hand coordinates because it implied a greater accuracy related to where to place the tip of the stylus on the surface, shifting α-DFA values toward 0.5. However, the hand orientation was not involved in this new task requirement, being possible to achieve the task goal using the same hand orientation than in the situation without the target or a variety of configurations to achieve the same result. That is, the target presence could cause a wide range of successful hand orientations which allow performing the final movement adjustments to place the tip of the stylus as close as possible to the target. The higher number of possible hand orientations to achieve the same outcome may reflect a higher flexibility to perform motion adjustments but not necessarily the requirement for performing them.

When we focused on the structure of the variability of hand orientation time series and its relationship with the amount of variability we found that without a target, participants who displayed a lower amount of variability showed hand orientation time series that were more autocorrelated. Following the previous idea, if participants did not have a reference to point with the stylus, similar configurations around a favourite hand orientation are possible, and exploration is not needed. On the other hand, those participants that used an imaginary reference would explore different hand orientations suitable to achieve that imaginary target, revealing higher autocorrelation values, considering that the differences are slight because there is no need for adjustment of this task dimension.

The MT was constrained during all the tasks by a metronome, which explains why autocorrelation of the variations in MT did not display significant differences according to the experimental conditions. This finding supports the previous idea that the effect of the task constraints depends on the task dimension the participant is trying to control. Interestingly, it showed an anti-persistent data distribution independently of the task performance with or without a target or reward. These results are quite different from those observed in self-paced movements in which, the structure of the MT shifted toward the pink noise region (α-DFA close to 1) [17,27]. Our results are in line with previous studies assessing variability structure of the MT during rhythmic manual tasks, which have found that external rhythm (i.e. metronome) led to white noise (α-DFA close to 0.5) or blue noise fluctuations (anti-persistent behaviour; α-DFA lower than 0.5) in MT time series [17,27,57,58]. These α-DFA values represent a fixed-point behaviour which confirms the individuals’ intention to synchronize their movement rhythm to the metronome stimulus.

## Conclusions

The performance of the task constrained with a target or target and reward required a higher implication or intentionality to adjust an individual’s movements to achieve the task goal, showing a decrease in long-range autocorrelation. This is to say individuals performed a greater number of adjustments to impact the target with highest accuracy. This aspect could be considered a sign of high alertness and attention, unfreezing degrees of freedom [12] of the motor system and allowing higher flexibility for movement adjustment. Nevertheless, the loss of relationship between variability structure and performance when the tasks were constrained could indicate different strategies of the participants to achieve the objective. Therefore, in light of our results and previous studies, this relationship between variability structure and performance could be dependent on task constraints such as feedback, experience, or difficulty or skill level of participants and it can be mediated by individual intrinsic constraints such as implication or intentionality to adjust to a target.

## Supporting information

S1 File Collected data. (XLSX)
